# Upper Limb Neural Tension Test and Spinal Biomechanics: Insights from a Longitudinal Pilot Study

**DOI:** 10.3390/bioengineering12050487

**Published:** 2025-05-02

**Authors:** Massimo Rossi, Marianna Signorini, Ali Baram, Mario De Robertis, Gabriele Capo, Marco Riva, Maurizio Fornari, Federico Pessina, Carlo Brembilla

**Affiliations:** 1 Scuolaecomskbo, Istituto Ortopedico Rizzoli, Via Giulio Cesare Pupilli 1, 40100 Bologna, Italy; massimo.rossi.md@gmail.com; 2 Dipartimento di Ingegneria Matematica, Politecnico di Milano, Via A.M. Ampere 2, 20133 Milan, Italy; marianna.signorini@gmail.com; 3 Department of Neurosurgery, IRCCS Humanitas Research Hospital, Via Alessandro Manzoni 56, 20089 Rozzano, Italy; ali.baram@humanitas.it (A.B.); mario.derobertis@humanitas.it (M.D.R.); gabriele.capo@humanitas.it (G.C.); marco.riva@hunimed.eu (M.R.); maurizio.fornari@humanitas.it (M.F.); federico.pessina@hunimed.eu (F.P.); 4 Department of Biomedical Sciences, Humanitas University, Via Rita Levi Montalcini 4, 20090 Pieve Emanuele, Italy

**Keywords:** Upper Limb Neural Tension Test, spine biomechanics, motion capture, least squares approximation, cervicobrachial neuralgia, manual therapy

## Abstract

**Background:** The Upper Limb Neural Tension Test (ULNTT) is a common assessment for neurodynamic function, yet the relationship between ULNTT findings and specific spinal biomechanical patterns remains poorly understood, particularly in the context of cervicobrachial neuralgia. This study aimed to investigate the association between ULNTT asymmetry and cervicothoracic spine biomechanics using advanced motion capture analysis. **Methods:** A longitudinal experimental study was conducted on two groups of asymptomatic participants: one with ULNTT asymmetry > 10° (AS group, n = 12) and another with symmetrical ULNTT (S group, n = 11). Neurodynamic testing and 3D motion capture of spinal kinematics during head lateral bending were performed at baseline. The AS group then underwent manual medicine intervention targeting spinal mobility impairments, followed by post-intervention reassessment. Spine biomechanics data, focusing on the C5-T4 region, were analyzed using the least squares approximation method to derive parameters describing upper thoracic (T1-T4_VERT) and lower cervical (C5-T1_CONC) lateral bending, and their interrelationship (ANGLE_TANG). **Results:** At baseline, the AS group showed significant differences between sides in neurodynamic parameters and T1-T4_VERT, with limited upper thoracic lateral bending contralateral to the side of the restricted ULNTT. Significant intergroup differences were also observed for these parameters. Following intervention in the AS group, significant improvements were noted in neurodynamic parameters and T1-T4_VERT, with no significant between-side differences post-intervention. **Conclusions:** These are preliminary results and preliminary conclusions based on the first study on a small group of patients. Given the limitations, this study provides evidence for a relationship between ULNTT asymmetry and upper thoracic spine biomechanics, specifically a contralateral limitation in lateral bending. These findings suggest a functional link between brachial plexus neurodynamics and upper thoracic spine mobility, offering potential insights into the pathophysiology of cervicobrachial conditions and highlighting the potential role of manual therapy in addressing both neurodynamic and biomechanical impairments. The developed motion capture analysis method offers a novel approach to quantify fine spinal motion patterns.

## 1. Introduction

The Upper Limb Neural Tension Test (ULNTT) is a widely used clinical tool for assessing the mobility and displacement of cervical nerve roots and the brachial plexus within their surrounding structures [1,2,3]. Characterized by good to excellent intra-tester reliability [4,5,6], it plays a crucial role in identifying the contribution of the nervous system to arm pain [7]. Alterations in neurodynamics, as detected by a positive ULNTT, are often implicated in the development of cervicobrachial neuralgia, a condition characterized by neuropathic pain in the neck and upper limbs [8,9].

In functional approaches to spine-related problems, such as osteopathy and chiropractic, the analysis of fine spinal motion patterns—subtle movements reflecting spinal function—is of paramount importance. However, a significant gap exists in the understanding of the relationship between specific spinal motion patterns and clinical conditions, particularly in the context of cervicobrachial neuralgia. Therefore, investigating the connection between ULNTT positivity and dysfunctional cervicothoracic spinal biomechanics is essential for elucidating the underlying pathophysiology and identifying potential therapeutic targets.

Traditional methods for studying spine biomechanics often rely on motion capture technology, which tracks the positional variations of marker clusters placed on anatomical landmarks [10,11,12,13,14]. While effective for capturing gross movements, these methods struggle to accurately quantify fine spinal motion patterns. Furthermore, as highlighted in recent studies, capturing spinal movements presents challenges due to the difficulty in identifying spinal joint centers with skin-mounted sensors, and the low amplitude of intervertebral motions [15,16]. To address these limitations, this study employs the least squares approximation method, a new advanced mathematical tool that enables a more precise and reliable analysis of motion capture data.

This longitudinal experimental study aims to explore and describe any potential relationship between ULNTT positivity and dysfunctional cervicothoracic spine biomechanics using motion capture technology enhanced by the least squares approximation method. By identifying and characterizing any potential relationship, we seek to provide new insights into the pathophysiology of cervicobrachial neuralgia and pave the way for more targeted therapeutic interventions. We hypothesize that ULNTT positivity will be associated with specific patterns of dysfunctional biomechanics in the cervicothoracic spine.

## 2. Materials and Methods

### 2.1. Study Design

This was a longitudinal pilot study.

### 2.2. Participants

Participants were recruited from students enrolled in the M.D. program at the University of Milano-Bicocca and patients attending the clinics of physicians specializing in musculoskeletal conditions who collaborated with the authors. Those who agreed to participate in the study underwent an eligibility assessment with the lead investigator. To ensure maximum reliability in testing, the same investigator conducted the ULNTT for all participants, considering the good to excellent intra-tester reliability of the test.

The study involved two groups of participants: one group with asymmetry observed during the ULNTT (AS group) and another group with symmetry during the same test (S group). Inclusion criteria for the AS group required a greater than 10° asymmetry on the ULNTT, while the S group included participants with symmetrical results on the same test. The threshold of 10° asymmetry was chosen based on previous studies of the ULNTT in asymptomatic individuals with arm pain, where asymmetry greater than this was considered significant [17,18,19,20]. Sample size calculations for the AS group were based on achieving 80% power (alpha = 5%) with a smallest detectable difference of 7° and a standard deviation of 10%, which is four times the typical value reported in the literature [21]. This resulted in a required sample size of 10, but we opted to include 12 participants for greater confidence in the results. For the S group, the sample size was also determined to achieve 80% power (alpha = 5%) based on preliminary data comparing differences between the two sides in parameters related to upper thoracic spine side bending. Given the novel motion capture method used, we selected a smallest detectable difference of 5°, although smaller differences were detectable when comparing clinical and instrumental measurements. With a standard deviation of 5°, the required sample size was 10, which was increased to 11 participants.

To focus on the biomechanical aspect of the study, only asymptomatic participants were included to exclude pain-related confounding factors in the ULNTT analysis. Participants were recruited for the study following an assessment of their eligibility through medical history and a targeted physical examination. Individuals were excluded if they had a history of trauma or surgery to the shoulder girdle, upper limb, or spine. Further exclusion criteria included the presence of peripheral neuropathies or central nervous system (CNS) diseases, a history of mental health disorders, and known allergies to plastic materials, latex, or adhesives. To ensure participant safety and the integrity of the study, individuals with a medical history or physical examination findings indicative of contraindications to spinal manipulation were also excluded, such as collagenopathies, elastopathies, suspected fracture, clinical signs of occipito-atlanto-axial instability identified through clinical tests, and bone metabolism disorders. Inclusion was also precluded for subjects with limited range of motion in any joint of the upper limb (shoulder, elbow, or wrist) and for individuals with a Body Mass Index (BMI) greater than 25, the latter criterion being motivated by the increased difficulty in accurately identifying the anatomical landmarks necessary for marker placement during motion capture acquisition. Participants were also excluded if they reported any pain in the upper quadrant of the body within the three months prior to enrollment, or in the presence of a diagnosis of rheumatoid arthritis or diabetes.

**Figure 1:**
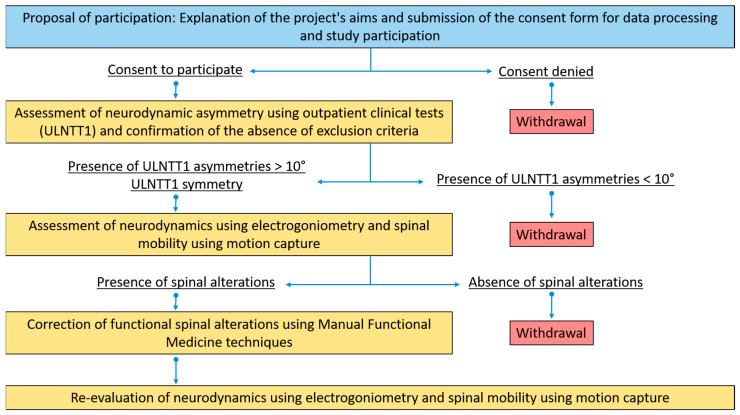
Study design flowchart.

The AS group consisted of 4 men and 8 women, with a median age of 30 years and a median BMI of 20.3. The S group consisted of 6 men and 5 women, with a median age of 27 years and a median BMI of 22.5. Participants who met the inclusion criteria and provided consent were evaluated at the Motion Capture Laboratory of the University of Milano-Bicocca, located at the Istituti Clinici Zucchi in Carate Brianza, where both neurodynamics and spine biomechanics were assessed using the ULNTT and motion capture techniques (Figure 1).

### 2.3. Neurodynamic Testing

**Figure 2:**
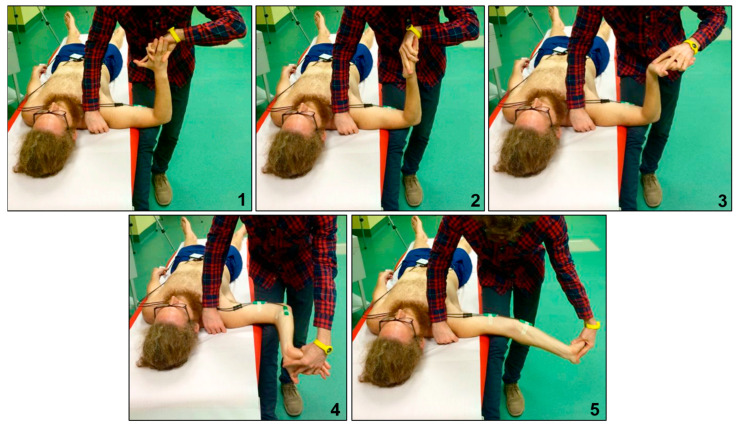
Performance of the ULNTT1, highlighting the 5 phases of the test. (**1**) Shoulder abduction to 90° and cranial stabilization of the scapular girdle with elbow flexion to 90°. (**2**) Forearm supination. (**3**) Wrist and first three fingers extension. (**4**) Shoulder lateral rotation to 90°. (**5**) Elbow extension, maintaining stabilization of the scapular girdle, distal arm, and wrist extension.

The ULNTT was performed according to the universally accepted procedure described by Butler [22] (Figure 2). The electrogoniometer used in the study was the Biometrics SG110 (Biometrics Ltd., Newport, UK). Two parameters were recorded during the test: resistance to elbow extension, referred to as R2 (operator-dependent), and the onset of severe pain, referred to as P2 (patient-dependent). To ensure accurate timing of P2, a light with a remote control was placed behind the electrogoniometer display. The patient was instructed to press a button on the remote control with their non-testing hand when severe pain was first perceived. This allowed the operator to continue testing without interruption and enabled the second operator to record the P2 value displayed. The P2 value was later reviewed by examining the recorded videos of the test. This method was believed to improve the intra-tester reliability for the R2 parameter, as it reduced potential interruptions during the test. To maintain the highest reliability in the ULNTT, all tests were conducted by the same operator, who remained blinded to the values displayed on the electrogoniometer. A second operator noted the readings. The test was performed four times per side, with a one-minute interval between repetitions to avoid increasing the range of motion (ROM), as reported in previous literature [4]. For the AS group, the first side tested was the one with the greater ROM in clinical evaluation to prevent conditioning that could affect the onset of symptoms.

### 2.4. Spine Mobility Testing

**Figure 3:**
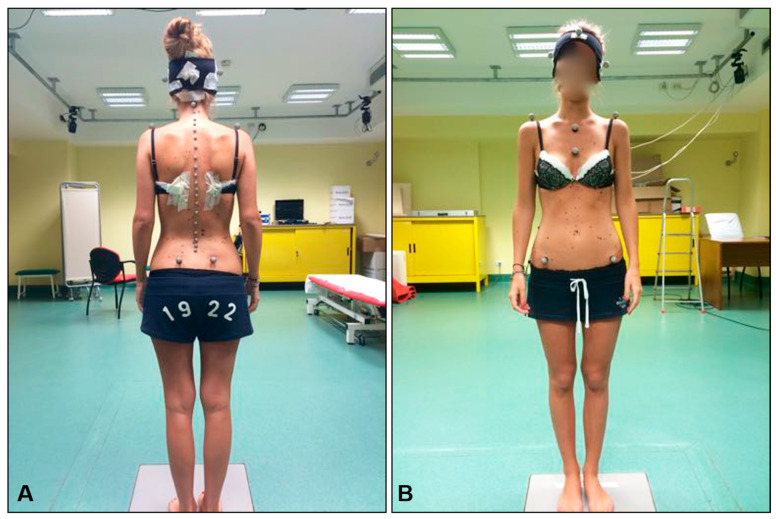
Marker placement for motion capture acquisition: (**A**) rear view, (**B**) front view.

Following the neurodynamic testing procedures, spine biomechanics were assessed using motion capture technology. Reflective markers were affixed to specific anatomical landmarks with double-sided adhesive tape, including C2, C5, C7, all spinous processes from T1 to L5, the acromions, the jugular notch, the xiphoid process, and the posterior and anterior superior iliac spines (Figure 3A). Additionally, a cap with four markers, two positioned on the sagittal plane and two on the frontal plane, was worn by each patient (Figure 3B). Movements were recorded by seven cameras (Qualisys Pro Reflex, Qualisys, Gothenburg, Sweden), four positioned posteriorly and three anteriorly relative to the patient.

The movement analyzed in this study was lateral bending of the head. Patients were positioned in front of a reference-less wall and instructed to maintain a forward gaze throughout the movement, continuing until they reached the end of the motion. Four repetitions were recorded for each side, alternating between the left and right sides, with the starting side chosen randomly.

### 2.5. Spine Intervention

At the conclusion of spine biomechanics assessments, patients with fine mobility impairments, defined as asymmetry in side bending during clinical testing, underwent manual medicine interventions aimed at correcting these impairments. The correction approach followed a functional perspective, treating the musculoskeletal system as an integrated unit. Due to the variability in patients’ specific biomechanical characteristics, no standardized treatment protocol was established.

The manipulative procedures performed on each patient are detailed in Table 1. These procedures included high-velocity, low-amplitude spinal manipulations and muscle energy techniques. The intervention lasted only a few minutes, and no spine marker except for C2 was removed during the intervention.

Immediately following the intervention, both spine biomechanics and neurodynamics were re-evaluated using the same methods as before.

### 2.6. Spine Biomechanics Data Analysis

The spine mobility data obtained using motion capture technology were analyzed using the least squares (LS) approximation method. This mathematical approach provides an analytical function (Equation (1)) that approximates discrete datasets without forcing the function to pass through the data points, as interpolation methods would. The decision to use approximation rather than interpolation was based on the nature of the dataset, which includes experimental measurements that may contain errors. Interpolation techniques could amplify these errors, leading to inconsistent results, while approximation avoids this issue.

**Figure 4:**
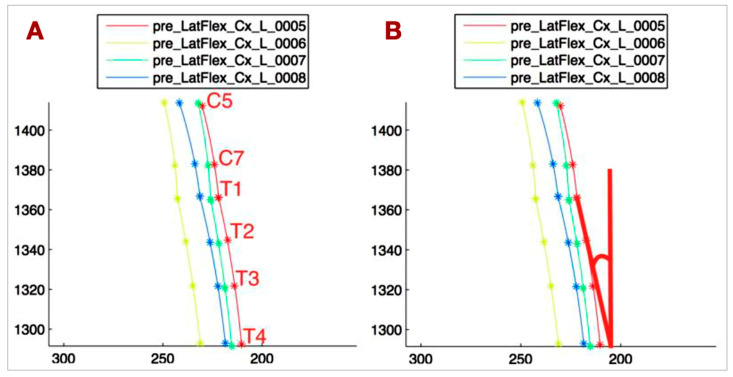
(**A**) Least squares approximation applied to markers on C5-T4 spinous processes during left head lateral flexion. Axes in millimeters. Four curves represent four movement repetitions. (**B**) Angle between T1-T4 curve tangent at T1 and vertical. Represents thoracic spine lateral movement and its contribution to cervical lateral flexion.

To approximate the shape of the spine, focusing on the regions from C5 to T4, a custom routine was developed in Matlab^®^ (version R2014, The MathWorks Inc., Natick, MA, USA). The spine was approximated in two segments: the first function approximated the coordinates of C5, C7, and T1, and the second approximated T1, T2, T3, and T4. This division was based on the distinct biomechanical patterns of the lower cervical and upper thoracic spine. The routine automatically identifies the maximum side bending of the spine. Figure 4A illustrates the results for patient number 1.

(1) $$\sum_{i=0}^{n}[y_{i}-\tilde{f}(x_{i}){]}^{2}\leq \sum_{i=0}^{n}{\left[y_{i}-p_{m}(x_{i})\right]}^{2}$$

**Figure 5:**
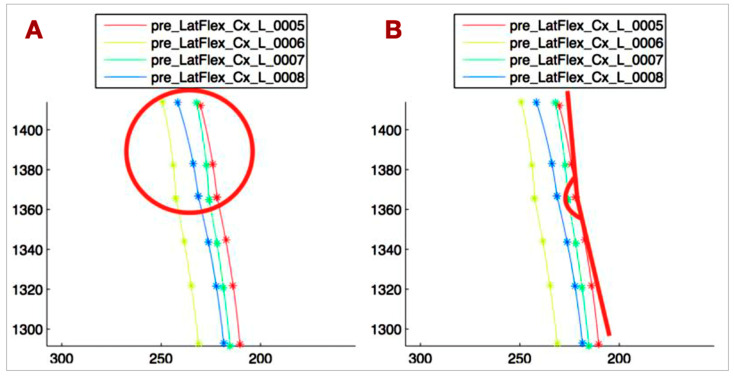
(**A**) C5-T1 curve concavity. Represents cervical spine lateral flexion magnitude. (**B**) Angle between T1-T4 and C5-T1 curve tangents at T1. Represents the relationship between cervical and thoracic spine curves.

These approximated functions were used to derive objective parameters that describe the biomechanics of the spine. The key parameters are as follows:

- T1-T4_VERT: The angle between the tangent at T1 to the T1-T4 curve and the vertical line, representing the side bending of the upper thoracic spine (Figure 4B).
- C5-T1_CONC: The C5-T1 concavity angle, which defines the side bending of C5-C7, independent of the upper thoracic side bending (Figure 5A).
- ANGLE_TANG: The angle between the tangent at T1 to the C5-T1 curve and the tangent at T1 to the T1-T4 curve, describing the relationship between the lower cervical and upper thoracic spine (Figure 5B).
- Head rotation (HEAD_ROT): The angular variation of the segment connecting the lateral markers on the head projected onto the horizontal plane, used to monitor head rotation. This parameter helps avoid bias in measuring spine lateral bending due to head rotation.

### 2.7. Statistical Analysis

All statistical analyses were conducted using IBM SPSS Statistics software (Version 22, IBM Corporation, Somers, NY, USA). The Wilcoxon Mann–Whitney test was used for comparison between groups. The level of significance for all statistical tests, including comparisons between pre- and post-intervention measures and between the two groups, was set at *p* < 0.05.

## 3. Results

The results are presented as medians in Table 2, Table 3 and Table 4. Angular measurements were used for all parameters, except C5-T1_CONC (1/m). P2 and R2 are reported in degrees, representing the difference in elbow extension between these points and full extension (0°); elbow hyperextension is expressed as a negative value.

### 3.1. Intragroup Comparisons at Baseline (Table 2)

- S group:
  Only R2 showed a statistically significant difference (2.5°), which is likely clinically insignificant and consistent with the literature on ULNTT asymmetry in asymptomatic individuals [17,18,19,20,23,24].
  No significant differences were observed in spine biomechanics parameters [25,26,27].
- AS group:
  ULNTT confirmed the inclusion criterion of >10° asymmetry (R2 median difference: 13.5°).
  P2 also showed a significant difference (14.5°), reinforcing R2 findings.
  T1-T4_VERT showed a significant difference, with 12.13° contralateral to the greater ULNTT ROM side and −1.17° contralateral to the smaller ROM side, indicating opposite T1-T4 movement to the cervical spine.
  No other spine biomechanics parameters, including HEAD_ROT, showed significant differences.

### 3.2. Intergroup Comparisons at Baseline (Table 3)

- Significant differences between S and AS groups were observed in R2 and P2 at the smaller ULNTT ROM side, and in T1-T4_VERT contralateral to the smaller ROM side.
- HEAD_ROT also differed significantly, but its clinical relevance is uncertain [28].
- R2 and P2 at the greater ULNTT ROM side, and T1-T4_VERT contralateral to the greater ROM side, were similar between groups.
- These findings suggest a relationship between upper limb neurodynamics and upper thoracic spine biomechanics.

### 3.3. Pre- to Post-Intervention Comparisons (AS Group—Table 4)

- Significant changes were observed in R2, P2, T1-T4_VERT, and C5-T1_CONC at the smaller ULNTT ROM side.
- These parameters were the same parameters that showed differences between groups at baseline.
- The clinical relevance of C5-T1_CONC changes is uncertain.

### 3.4. Post-Intervention Intragroup Comparisons (AS Group—Table 5)

No significant differences were observed between sides in any parameters post-intervention.

### 3.5. Data Interpretation

The results indicate a potential association between ULNTT findings and cervicothoracic spine biomechanics. Particularly, the T1-T4_VERT parameter consistently showed significant differences related to ULNTT asymmetry and intervention effects. The observed changes in neurodynamic parameters (R2, P2) alongside T1-T4_VERT support a functional link between upper limb neurodynamics and upper thoracic spine mobility. The lack of significant differences in other spine biomechanics parameters suggests that the relationship may be specific to T1-T4 lateral bending. The elimination of significant differences between sides post-intervention in the AS group highlights the potential impact of manual therapy on both neurodynamic and biomechanical parameters.

## 4. Discussion

This research addresses a gap in the current understanding of brachial plexus pathophysiology, specifically concerning the altered interface with surrounding structures. There is a notable absence of data linking clinical functional spinal alterations to specific organic diagnoses, particularly brachial plexopathy, and a lack of methods to objectively quantify these functional changes [19,21,24,27]. Moreover, effective therapeutic strategies for this condition are limited.

Our study’s findings reveal that neurodynamic parameters and the angle between the T1-T4 tangent at T1 and the vertical line exhibit statistically significant differences within the AS group, but not within the S group. These parameters, reflecting both neurodynamics and upper thoracic spine biomechanics, show significant intergroup differences at the side of restricted neurodynamics and contralateral to this side for spinal motion. Furthermore, these parameters improve significantly following functional correction and show no significant differences post-intervention.

Based on these observations, it is plausible to suggest, within the limitations of our study, that a relationship between brachial plexus neurodynamics and thoracic spine mobility may exist. Specifically, this relationship manifests as a limitation in thoracic spine lateral bending contralateral to the brachial plexus neurodynamic restriction.

The limitation in upper thoracic spine lateral flexion may reduce the contribution of the thoracic spine to the overall lateral bending movement, potentially leading to an increased range of motion demand on the cervical spine and subsequent tension on contralateral paravertebral cervical musculature, particularly the anterior and middle scalene muscles. Given their insertion on the first rib, which is mechanically linked to T1, increased tension in these muscles could alter their relationship with the brachial plexus, which courses between them, thereby limiting its mobility.

Nerve structure sensitization due to increased mechanical stress on the brachial plexus during daily head movements, mediated by the scalene muscles, may predispose patients to cervicobrachialgia in conjunction with other factors like trauma or adverse environmental conditions such as poor workplace ergonomics or repetitive upper limb strain [14,15].

Our study’s sample size limits the assessment of the prevalence of thoracic spine lateral flexion limitations contralateral to neurodynamic restrictions. Although the observed limitation in 11 out of 12 AS group patients suggests a potential association, larger studies are needed to contextualize its role in the pathophysiological process. Nevertheless, our findings establish a relationship between this limitation and neurodynamics. This connection aligns with the conceptual premise of functional diagnosis, emphasizing the importance of biomechanical assessment in neuromusculoskeletal symptom management.

Traditional approaches to spine biomechanics analysis, often employing motion capture systems that track skin-mounted markers [10,11,12,13,14], are known to have limitations in accurately quantifying subtle spinal movements. As highlighted by the recent literature [15,16], the challenges arise from difficulties in precisely locating spinal joint centers with external sensors and the inherently small amplitude of intervertebral motion. In contrast, our study utilized the least squares approximation method, an advanced mathematical tool allowing for a more precise and reliable analysis of the motion capture data we acquired. These methods offer the advantage of analyzing the contribution of individual vertebrae to spinal mechanics, though they are limited to single-plane analysis and require specialized mathematical expertise. Consequently, their application to the upper and mid-cervical spine, characterized by complex coupled movements, is restricted. For lumbar spine analysis, vertical planes through the posterior superior iliac spines may mitigate pelvic girdle rotation effects [15,16]. Multiplanar motion analysis is feasible with these methods but requires separate assessments. Finally, the time required for data acquisition and analysis and the need for specialized personnel may limit their broader research application.

Several limitations inherent in this preliminary study warrant careful consideration when interpreting these findings. The study’s limitations included a small sample size, which inherently restricts the generalizability of our results to a larger population. The lack of randomization in participant selection may have introduced potential biases that could influence the observed associations. The analysis of spinal motion was limited to a single plane, specifically lateral bending, which may not fully capture the complex interplay between ULNTT asymmetry and overall cervicothoracic biomechanics. Additionally, the manual therapy interventions were not standardized in this study; each participant received individually selected techniques, which limits the comparability of the results and their reproducibility in other studies. Further clinical studies in patients with cervicobrachialgia due to altered brachial plexus interface are necessary to evaluate the therapeutic potential of correcting functional spinal alterations, particularly in those with upper thoracic spine lateral flexion limitations contralateral to the plexopathy. Future research should aim to address these limitations by employing larger, randomized controlled trials and by investigating spinal motion in multiple planes, alongside standardized therapeutic interventions, to provide more robust and generalizable conclusions.

## 5. Conclusions

In conclusion, our findings support the hypothesis that a relationship exists between the Upper Limb Neural Tension Test (ULNTT) and spine biomechanics. Specifically, this relationship is characterized by a limitation in upper thoracic spine lateral bending on the side contralateral to the restricted ULNTT in patients exhibiting asymmetry greater than 10 degrees. This observation underscores the biomechanical interplay between spinal mobility and neurodynamics, contributing insights for future research and the possible development of targeted therapeutic approaches. By highlighting this connection, we open avenues to explore more effective interventions for patients presenting ULNTT asymmetry and related spinal biomechanical alterations. It should be emphasized that these are preliminary results, based on a study of a small group of patients. Further research with a larger patient population is needed to confirm these preliminary results, including symptomatic individuals.

## Figures and Tables

**Table 1:** Treatments applied to the patients in group PZ; hvla: high-velocity low-amplitude spinal manipulations; mit: Mitchell techniques. Letters N/F/E, S, R following each vertebra: primary direction of motion of the vertebra in the sagittal plane (N: no primary direction; F: increased range of motion in flexion; E: increased range of motion in extension), frontal plane (S left: increased side bending to the left; S right: increased side bending to the right), and horizontal plane (R left: increased rotation to the left; R right: increased rotation to the right).

PZ1	hvla L3 N S left R left	PZ8	hvla L3 N S left R left
	mit T9-L1 ERS right		mit T9-L1 ERS right
			mit T9-L1 ERS right
PZ3	hvla L4 N S left R left	PZ9	hvla L3 N S left R left
	mit T8-T12 ERS right		mit T8-T12 ERS right
			hvla C5 N S left R left
PZ4	hvla L3 N S left R left	PZ10	hvla L4 N S left R left
	mit T9-L1 ERS right		mit T9-L1 ERS right
	hvla C5 N S left R left		hvla C6 N S left R left
			mit T7-T9 ERS right
PZ5	hvla L3 N S left R left	PZ11	hvla L3 N S left R left
	mit T10-L1 ESR left		mit T9-L1 ERS left
	hvla C6 N S right R right		hvla C6 N S left R left
PZ6	hvla L3 N S left R left	PZ12	hvla L3 N S right R right
	mit T9-T12 ERS right		mit T10-L1 ERS left
	hvla C5 N S left R left		hvla C5 N S left R left
PZ7	hvla L4 N S left R left	PZ13	hvla L4 N S right R right
	mit T9-L1 ERS right		mit T9-L1 ERS right
	hvla C6 N S left R left		hvla C6 N S left R right

**Table 2:** Intragroup parameters comparison at baseline.

Null Hypothesis	Group	*p* Value	Median (Min/Max) Greater ROM Side	Median (Min/Max) Smaller ROM Side
Median of following parameter differences equals zero				
R2 greater ROM side/R2 smaller ROM side	S	0.003 *	−2.25 (−7.25/3.75)	0.25 (−7.00/5.75)
	AS	0.002 *	0.12 (−7.25/6.25)	13.87 (7.25/23.25)
P2 greater ROM side/P2 smaller ROM side	S	0.123	0.00 (−7.25/12.00)	0.5 (−7.00/12.00)
	AS	0.002 *	3.12 (−5/7.75)	17.62 (9.26/28.00)
T1-T4_VERT controlat ULNTT greater ROM side/T1-T4_VERT controlat ULNTT smaller ROM side	S	0.306	11.72 (2.68/24.37)	12.73 (5.38/22.22)
	AS	0.003 *	12.13 (4.70/19.83)	−1.17 (−3.90/7.63)
C5-T1_CONC controlat ULNTT greater ROM side/C5-T1_CONC controlat ULNTT smaller ROM side	S	0.824	0.0029 (−0.0025/0.0086)	0.0024 (0.0006/0.0056)
	AS	0.195	0.0022 (0.0003/0.112)	0.0026 (−0.001/0.0083)
ANGLE_TANG controlat ULNTT greater ROM side /ANGLE_TANG controlat ULNTT smaller ROM side	S	0.722	4.34 (0.05/9.78)	4.6 (1.55/6.83)
	AS	0.077	5.73 (1.50/15.51)	3.26 (0.13/18.02)
ROT_CERV controlat ULNTT greater ROM side/ROT_CERV controlat ULNTT smaller ROM side	S	1	12.45 (2.81/46.42)	17.05 (7.44/24.64)
	AS	0.099	12.74 (3.97/31.48)	6.51 (2.18/18.42)

* means that that value has statistical significance *(p* < 0.05).

**Table 3:** Intergroup parameters comparison at baseline.

Null Hypothesis	*p* Value	Median (Min/Max) S	Median (Min/Max) AS
Parameter distribution is the same in S and AS groups			
R2 greater ROM side	0.104	−2.25 (−7.25/3.75)	0.12 (−7.25/6.25)
R2 smaller ROM side	<0.001 *	0.25 (−7.00/5.75)	13.87 (7.25/23.25)
P2 greater ROM side	0.535	0.00 (−7.25/12.00)	3.12 (−5/7.75)
P2 smaller ROM side	<0.001 *	0.5 (−7.00/12.00)	17.62 (9.26/28.00)
T1-T4_VERT controlat ULNTT greater ROM side	1	11.72 (2.68/24.37)	12.13 (4.70/19.83)
T1-T4_VERT controlat ULNTT smaller ROM side	<0.001 *	12.73 (5.38/22.22)	−1.17 (−3.90/7.63)
C5-T1_CONC controlat ULNTT greater ROM side	0.695	0.0029 (−0.0025/0.0086)	0.0022 (0.0003/0.112)
C5-T1_CONC controlat ULNTT smaller ROM side	0.695	0.0024 (0.0006/0.0056)	0.0026 (−0.001/0.0083)
ANGLE_TANG controlat ULNTT greater ROM side	0.487	4.34 (0.05/9.78)	5.73 (1.50/15.51)
ANGLE_TANG controlat ULNTT smaller ROM side	0.651	4.6 (1.55/6.83)	3.26 (0.13/18.02)
ROT_CERV controlat ULNTT greater ROM side	0.976	12.45 (2.81/46.42)	17.05 (7.44/24.64)
ROT_CERV controlat ULNTT smaller ROM side	0.007 *	12.74 (3.97/31.48)	6.51 (2.18/18.42)

* means that that value has statistical significance *(p* < 0.05).

**Table 4:** AS pre/post-intervention parameters comparison.

Null Hypothesis	*p* Value	Median (Min/Max) Pre	Median (Min/Max) Post
Median of following parameter differences equals zero			
R2 greater ROM side pre/R2 greater ROM side post	0.182	0.12 (−7.25/6.25)	−1.62 (−8.00/4.00)
R2 smaller ROM side pre/R2 smaller ROM side post	0.002 *	13.87 (7.25/23.25)	−0.12 (−8.25/5.75)
P2 greater ROM side pre/P2 greater ROM side post	0.109	3.12 (−5/7.75)	0.62 (−8.00/6.75)
P2 smaller ROM side pre/P2 smaller ROM side post	0.002 *	17.62 (9.26/28.00)	1.87 (−8.25/5.75)
T1-T4_VERT controlat ULNTT greater ROM side pre/ T1-T4_VERT controlat ULNTT greater ROM side post	0.638	12.13 (4.70/19.83)	9.84 (6.52/20.68)
T1-T4_VERT controlat ULNTT smaller ROM side pre/ T1-T4_VERT controlat ULNTT smaller ROM side post	0.003 *	−1.17 (−3.90/7.63)	7.33 (3.11/14.58)
C1-C5_CONC controlat ULNTT greater ROM side pre/ C1-C5_CONC controlat ULNTT greater ROM side post	0.41	0.0022 (0.0003/0.112)	0.0023 (0.0003/0.0010)
C1-C5_CONC controlat ULNTT smaller ROM side pre/ C1-C5_CONC controlat ULNTT smaller ROM side post	0.034 *	0.0026 (−0.001/0.0083)	0.0043 (0.0005/0.0010)
ANGLE_TANG controlat ULNTT greater ROM side pre/ANGLE_TANG controlat ULNTT greater ROM side post	0.754	5.73 (1.50/15.51)	4.71 (2.51/14.80)
ANGLE_TANG controlat ULNTT smaller ROM side pre/ANGLE_TANG controlat ULNTT smaller ROM side post	0.754	3.26 (0.13/18.02)	4.01 (1.96/10.87)
CONC_CERV controlat ULNTT greater ROM side pre/CONC_CERV controlat ULNTT greater ROM side post	0.117	17.05 (7.44/24.64)	15.82 (1.73/38.73)
CONC_CERV controlat ULNTT smaller ROM side pre/CONC_CERV controlat ULNTT smaller ROM side post	0.308	6.51 (2.18/18.42)	6.69 (2.66/25.50)

* means that that value has statistical significance *(p* < 0.05).

**Table 5:** AS group post-intervention parameters comparison.

Null Hypothesis	*p* Value	Median (Min/Max) Greater ROM Side	Median (Min/Max) Smaller ROM Side
Median of following parameter differences equals zero			
R2 greater ROM side/R2 smaller ROM side	0.789	−1.62 (−8.00/4.00)	−0.12 (−8.25/5.75)
P2 greater ROM side/P2 smaller ROM side	0.755	0.62 (−8.00/6.75)	1.87 (−8.25/5.75)
T1-T4_VERT controlat ULNTT greater ROM side/T1-T4_VERT controlat ULNTT smaller ROM side	0.158	9.84 (6.52/20.68)	7.33 (3.11/14.58)
C5-T1_CONC controlat ULNTT greater ROM side/C5-T1_CONC controlat ULNTT smaller ROM side	0.13	0.0023 (0.0003/0.0010)	0.0043 (0.0005/0.0010)
ANGLE_TANG controlat ULNTT greater ROM side /ANGLE_TANG controlat ULNTT smaller ROM side	0.99	4.71 (2.51/14.80)	4.01 (1.96/10.87)
ROT_CERV controlat ULNTT greater ROM side/ROT_CERV controlat ULNTT smaller ROM side	0.209	15.82 (1.73/38.73)	6.69 (2.66/25.50)

## Data Availability

The corresponding author will share the data upon request due to legal and ethical reasons.
